# Dynamic Culture Substrates That Mimic the Topography of the Epidermal–Dermal Junction

**DOI:** 10.1089/ten.tea.2018.0125

**Published:** 2019-02-11

**Authors:** Ayelen L. Helling, Priyalakshmi Viswanathan, Katerina S. Cheliotis, Seyedeh Atefeh Mobasseri, Ying Yang, Alicia J. El Haj, Fiona M. Watt

**Affiliations:** ^1^ Center for Stem Cells and Regenerative Medicine, King's College London, Guy's Hospital, London, United Kingdom.; ^2^ Institute for Science and Technology in Medicine, Keele University, Stoke-on-Trent, United Kingdom.

**Keywords:** human epidermal–dermal junction, dynamic substrate, keratinocytes

## Abstract

The junction between the epidermal and dermal layers of human skin undulates, the width and depth of the undulations varying with age and disease. We previously showed that when primary human epidermal keratinocytes are seeded on collagen-coated undulating static polydimethylsiloxane (PDMS) elastomer substrates, the stem cells, differentiated cells, and proliferating cells become patterned in response to cues from the underlying substrate. To investigate how patterning occurs over time, we have now created a dynamic model, in which a collagen-coated poly(d,l-lactide-co-glycolide) (PLGA) membrane is placed over a polyimide sheet containing circular holes, differing in diameter and spacing. When a vacuum is applied the membrane is induced to undulate, the heights of the undulations depending on the pressure applied and the size of the holes. We observed clustering of cells with high levels of β1 integrin expression, a stem cell marker, in the base of the undulations within 48 h of applying the vacuum. Differentiating involucrin-positive cells did not cluster; however, there was clustering of cells with high E-cadherin expression and nuclear YAP. Rho kinase inhibition resulted in loss of clustering, suggesting a role for Rho family members in the process.

**Impact Statement:**

In human skin the junction between the epidermis and dermis undulates. Epidermal stem cells pattern according to their position relative to those undulations. Here we describe a rig in which epidermal cells are cultured on a collagen-coated poly(d,l-lactide-co-glycolide) (PLGA) membrane. When a vacuum is applied the membrane is induced to undulate. Stem cells cluster in response to the vacuum, whereas differentiating cells do not. Rho kinase inhibition results in loss of clustering, suggesting a role for Rho family members in the process. This dynamic platform is a new tool for investigating changes in the skin with age and disease.

## Introduction

Mammalian skin is a complex organ that consists of two major layers, the epidermis and the dermis, separated by a basement membrane that is rich in type IV collagen and laminin.^1^ The structure of the epidermal–dermal junction in human skin is not flat but undulates, forming a pattern of alternating rete ridges, where the epidermis projects more deeply into the dermis, and dermal papillae, where the dermis comes closest to the skin surface.^2^ The depth and width of the rete ridges vary with age and body site and are increased in inflammatory skin diseases such as psoriasis.^3–8^

Stem cells are located in the human epidermal basal layer^9,10^ and occupy specific locations relative to the epidermal–dermal junction, in most body sites clustering on the top of the dermal papillae.^9,11,12^ Expression of several cell surface markers is enriched in human epidermal stem cells, including β1-integrin receptors, Lrig1, CD46, MCSP, and Delta-like 1.

Previous studies have demonstrated that stem cell fate is regulated by a combination of intrinsic (genetic and epigenetic) and extrinsic signals,^1,13^ such as soluble factors, cell–cell contact, extracellular matrix (ECM) protein interactions, and tissue topography. To mimic different components of the extracellular environment, a number of approaches have been taken, such as coculture of keratinocytes and dermal fibroblasts on biomaterials, including collagen gels,^14^ which act as scaffolds for cell growth and also facilitate cell–cell interactions. Other approaches include culture on de-epidermized acellular human dermal matrices,^15^ self-assembled living sheets made with human fibroblasts and keratinocytes,^16^ and bioprinted cell-laden hydrogels.^17^ However, these models fail to mimic the undulating epidermal–dermal junction. To overcome this limitation several other models^18–20^ have been developed. Undulations have been created using static topographies through microfabrication of dermal–epidermal regeneration matrices, for example, through fabrication of patterned polydimethylsiloxane (PDMS) substrates or micro-topographies using photolithography followed by the production of collagen-GAG templates.^19^

Using a panel of undulating collagen-coated PDMS substrates^18^ that differ in diameter, height, and center-to-center spacing, we have previously shown that topography is sufficient to direct the formation of β1 integrin bright stem cell clusters on the top of the features. In addition, we found that separate topographical cues determine the locations of stem cells, involucrin-positive differentiated cells, and proliferating cells. This prompted us to develop a platform in which we could change topography from flat to undulating to measure dynamic cell responses. By simultaneously creating multiple topographies within a single device, we can begin to understand the effect of aging and other changes on stem cell behavior.

## Materials and Methods

### Membrane preparation

Poly(d,l-lactide) (PLA, average Mw 75,000–120,000, ref P1691) and poly(d,l-lactide-co-glycolide) (PLGA, average Mw 50,000–75,000, ref 430471) were purchased from Sigma-Aldrich. Dichloromethane (DCM, anhydrous, ≥99.8% ref 270997) was purchased from Sigma-Aldrich and was used as a solvent to prepare the PLA and PLGA membranes. PLA was dissolved in DCM in a 5 $${ \raise0.7ex \hbox{${ \rm{w}}$}   \mathord{ \left/ { \vphantom
{{ \rm{w}} { \rm{v}}}} \right. \kern- \nulldelimiterspace}
\lower0.7ex \hbox{${ \rm{v}}$}}{ \rm{ \% }}$$ and PLGA in a 2 $${\raise0.7ex \hbox{${ \rm{w}}$}   \mathord{ \left/ { \vphantom {{
\rm{w}} { \rm{v}}}} \right. \kern- \nulldelimiterspace}
\lower0.7ex \hbox{${ \rm{v}}$}}{ \rm{ \% }}$$, using the formula:

\begin{align*}
{ \raise0.7ex \hbox{${ \rm{w}}$}   \mathord{ \left/ { \vphantom {{
\rm{w}} { \rm{v}}}} \right. \kern- \nulldelimiterspace}
\lower0.7ex \hbox{${ \rm{v}}$}}{ \rm{ \% = }}{ \raise0.7ex
\hbox{${{ \rm{mass \;of \;polymer \;}} \left( { \rm{g}} \right)
}$}   \mathord{ \left/ { \vphantom {{{ \rm{mass \;of \;polymer
\;}} \left( { \rm{g}} \right) } {{ \rm{volume \;solvent \;}}
\left( {{ \rm{mL}}} \right) }}} \right. \kern-
\nulldelimiterspace}   \lower0.7ex \hbox{${{ \rm{volume \;solvent
\;}} \left( {{ \rm{mL}}} \right) }$}}{ \rm{ \times 100}} \tag{1}
\end{align*}

The concentrations were chosen based on the lowest amount of polymer able to form an elastic thin membrane that will deform under vacuum without collapsing. The solution was made in a glass vial and gently agitated using a magnet stirrer for ∼1 h. After complete dissolution, 7 mL of the solution was placed on a 13.5 cm diameter glass Petri dish and covered with a lid to guarantee slow evaporation of the solvent at room temperature for 48 h inside the fume hood. Samples were then sterilized by covering the surface with 70% ethanol for 30 min at room temperature.

The melting point of the two membranes (PLGA and PLA) was measured using a Differential Scanning Calorimeter (DSC)-Mettler Toledo DSC822e Calorimeter. Three samples for each polymer were analyzed. Sample size was 10 ± 1 mg.

The tensile strength of the membranes was measured using uniaxial tensile test—ElectroForce Model 3200 testing machine (BOSE). The samples were cut according to the ASTM D882 Tensile Strength properties of thin plastic films (film less than 1 mm thick, in a ratio 2:1; 3:0.5 inch). The samples were 10 × 30 mm in dimension, tested with a maximum load of 22.2 N and a rate of 0.05 mm/s.

### Collagen coating

PLGA membranes were coated with collagen type I (Ref 354236; Corning) in phosphate-buffered saline (PBS) for 2 h at 37°C. To evaluate collagen deposition membranes were labelled with anti-Collagen I antibody (1:500, ab34710; Abcam) followed by donkey anti-rabbit 488 Alexa Fluor secondary antibody (1:1000, A-21206; Thermo Fisher). Collagen coated and noncoated membranes were then imaged by confocal microscopy.

### Template design

Three topographies were designed using the software AutoCAD Autodesk 2016. The topographies were distributed according to the shape of a 12-well cell culture plate to cover surfaces of 1 cm by 1 cm. Each of the three topographies and a flat control were arranged in triplicate. The dimensions that differed between topographies were the diameters of the drilled holes and the distance between holes. The first topography consisted of drilled holes with a diameter of 100 μm and a distance between holes of 150 μm. The second topography consisted of drilled holes with a diameter of 100 μm and interhole distance of 200 μm. The third topography had holes of 200 μm in diameter and a distance of 200 μm between holes.

The template material selected was a 500 μm thick polyimide sheet (Cirlex from Goodfellow), which has good mechanical properties and yet is thin enough for laser drilling. Laser drilling requires a small ratio diameter of drilling: sheet thickness, because the drilled holes become conical if the thickness is too high.

### Rig design

The rig was designed using AutoCAD Autodesk 2016 and fabricated with transparent acrylic. It was based on the dimensions of a 12-well cell culture plate and composed of three individual parts that are assembled together and secured with screws. The rig has two barbed fittings to connect the pipe that is attached to the vacuum pump (SAM 18, MGE) that generates the force to deform the culture membrane. The manufacturing process was carried out by the Department of Clinical Technology Mechanical Workshop Activity of the University Hospital of North Midlands NHS Trust.

### Scanning electron microscopy

Scanning electron microscopy (SEM) was used to evaluate the deformation of membranes in the presence of a vacuum. To fabricate the samples for SEM, PDMS was added on the membrane while the vacuum was running. Following complete curing, PDMS was peeled from the membrane, creating a replica of the patterns of the membrane. Samples were coated with gold sputter (4 nm thickness) and imaged with SEM using a JEOL NeoScope JCM 6000Plus.

### Culture of human keratinocytes

Neonatal human foreskin keratinocytes were cultured on a mitotically inactivated feeder layer comprising the J2 clone of 3T3 cells. As described previously^18^ the medium comprised 1 part Ham's F12, 3 parts Dulbecco's modified Eagle's medium, 1.8 × 10^−4^ M adenine, 10% (v/v) fetal bovine serum (FBS), 0.5 μg/mL hydrocortisone, 5 μg/mL insulin, 10^−10^ M cholera toxin, and 10 ng/mL epidermal growth factor [complete F12, Adenine, and DMEM (FAD) medium]. Keratinocytes were seeded onto PLA and PLGA membranes at a density of 100,000 cells/cm^2^ overnight in complete Keratinocyte Serum Free Medium (KSFM; Gibco) and then transferred for 48 h to complete FAD medium at 37°C and 5% CO_2_. No vacuum pressure was applied to the rig during the first 20 min in FAD medium. Following this, constant vacuum pressure was applied through the rig, deforming the membrane on which keratinocytes were growing.

### Immunolabeling and confocal microscopy

Cells were simultaneously fixed and permeabilized in 4% paraformaldehyde (PFA) and 0.2% Triton X-100 (Sigma) for 15 min, at room temperature. Afterwards they were blocked for 1 h in blocking buffer (10% FBS, 0.25% gelatin from cold water fish skin [Sigma] in PBS) at room temperature, incubated with primary antibodies diluted in blocking buffer for 1 h at room temperature or overnight at 4°C, and incubated with Alexa Fluor (488 and 555)-conjugated secondary antibodies diluted in PBS for 1 h at room temperature. Samples were mounted using mounting medium with DAPI (ProLong Gold antifade reagent with DAPI; Invitrogen). The following primary antibodies were used: P5D2 (mouse monoclonal anti-β1 integrin; dilution of 1:500; prepared in-house); SY7 (mouse monoclonal anti-involucrin; dilution of 1:1000; prepared in-house); HECD-1 (mouse monoclonal anti-E-cadherin; dilution of 1:1000; prepared in-house); and anti-YAP (rabbit; dilution of 1:200; Cell Signaling). Samples were visualized using a Nikon A1 Scanning Confocal Upright microscope.

### Statistical analysis

All values are represented as mean ± standard deviation. Experiments were performed in triplicate. Additional information is provided in the figure legends. Statistical analysis was carried out using GraphPad Prism 7.0 (GraphPad Software, Inc.). All data were analyzed by one or two factor ANOVA tests. The statistical significance was set at *p < 0.05, **p < 0.01, ***p < 0.001, and ****p ≤ 0.0001.

## Results

### Membrane characterization

**FIG. 1.**
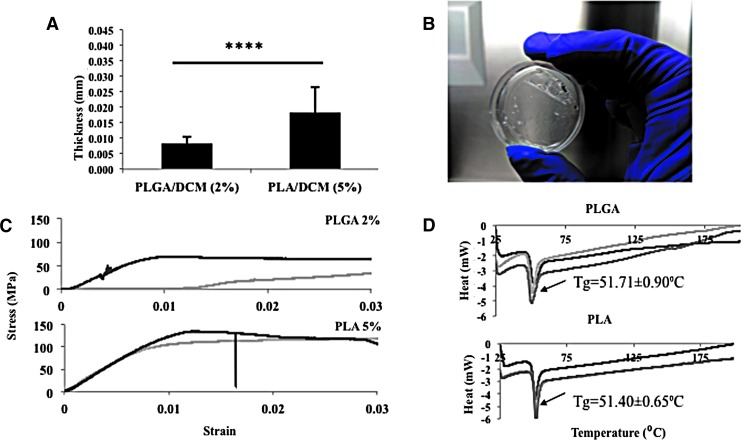
Membrane characterization. **(A)** Thickness of 2% (w/v) PLGA and 5% (w/v) PLA dissolved in DCM using the same volume of solvent. ****p ≤ 0.0001. **(B)** Photograph illustrating the transparency of the PLGA membrane. **(C)** Mechanical characterization using uniaxial tensile test of PLGA and PLA. **(D)** Differential scanning calorimeter test of 2% (w/v) PLGA and 5% (w/v) PLA to determine the melting point of each polymer. The multiple curves are replicate tests. **(A, C, D)** Three samples of each polymer were analyzed. DCM, dichloromethane; DSC, differential scanning calorimeter; PLA, poly(d,l-lactide), PLGA, poly(d,l-lactide-co-glycolide).

PLA and PLGA membranes have been used extensively to support the growth of cultured cells and are translucent, enabling cells to be visualized by light microscopy.^21,22^ The polymer concentration of each membrane was chosen based on the minimum concentration that could create an elastic thin membrane that would deform under vacuum pressure without collapsing: 2% (w/v) PLGA and 5% (w/v) PLA. We therefore compared their thickness and mechanical properties with a view to selecting one as the material for the dynamic substrate. The thickness of the membranes was measured using a digital micrometer (Fig. 1A, B). We also subjected the membranes to a uniaxial tensile test (Fig. 1C) to compare the elasticity and breaking strength of the materials. Finally, we measured crystallinity using DSC (Fig. 1D): PLGA and PLA had a similar melting point, which was higher than 37°C, allowing us to conclude that culturing cells would not affect the chemical properties of either polymer. Based on these results, PLGA was chosen over PLA because of its lower thickness and higher elasticity.

### Template design

**FIG. 2.**
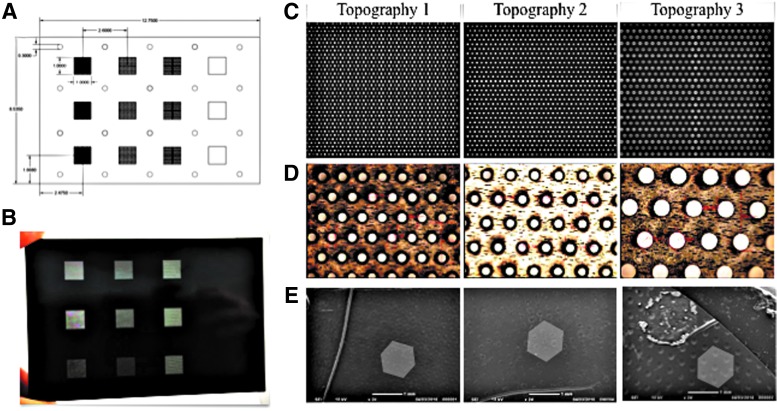
Template design and characterization. **(A)** Design of the template in AutoCAD. **(B)** Template produced in a 500 μm thick polyimide sheet. **(C, D)** Light microscopy of each topography in the polyimide sheet, showing diameter and spacing of holes. Area of each sheet in **(C)** is 1 × 1 cm. **(E)** SEM images of PLGA membrane placed on top of the topographies after applying 20 kPa vacuum pressure for 30 min. Scale bars in **(E)** are 1 mm. SEM, scanning electron microscopy.

The template for deforming the PLGA membrane was designed using AutoCAD software (Fig. 2A) and fabricated by laser drilling a 500 μm thick polyimide sheet (Fig. 2B). The dimensions of each feature were selected based upon previous studies elucidating epidermal–dermal junction topographies in young and aged skin.^5^ Three topographies were designed, each of which covered the area of an individual well of a 12-well plate. We also included a fourth topography with no holes, to serve as a flat membrane control. Each topography was reproduced thrice in the template, so that within a single experimental run we could compare triplicate cultures exposed to each topography (Fig. 2B–E). As shown in Figure 2C–E, there was excellent uniformity in the size and patterning of the holes.

### Rig design and assembly

**FIG. 3.**
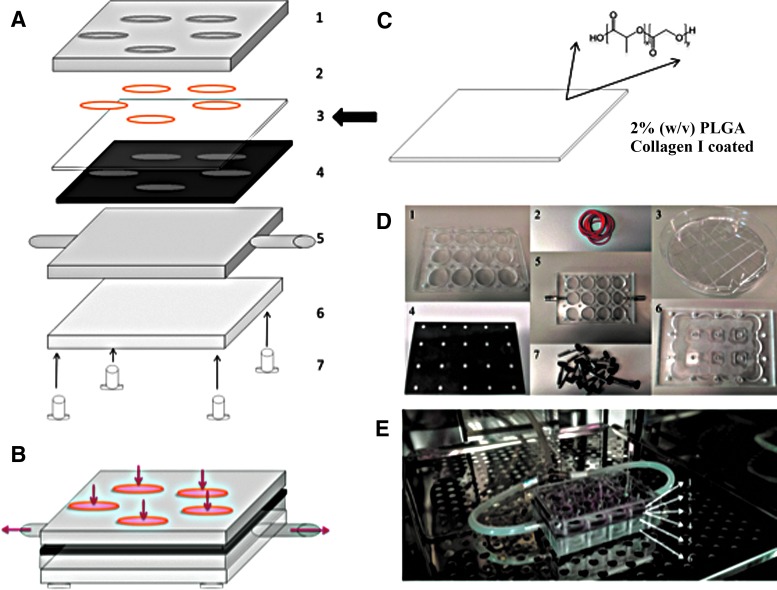
Schematic diagram of rig-template-membrane assembly. **(A)** The polyimide template (4) is placed on top of that part of the rig with the outlets to the vacuum pump (5). The PLGA membrane (3) is placed on top of the template and is deformed when vacuum suction is applied. The *arrow* indicates where the component in panel C is incorporated into the overall structure shown in A. **(B)**. The other components of the assembly **(A)** are shown in **(D)**. **(C)** PLGA is precoated with Collagen I before assembly of the rig. **(E)** The different components **(A, D)** in an incubator following assembly.

**FIG. 4.**
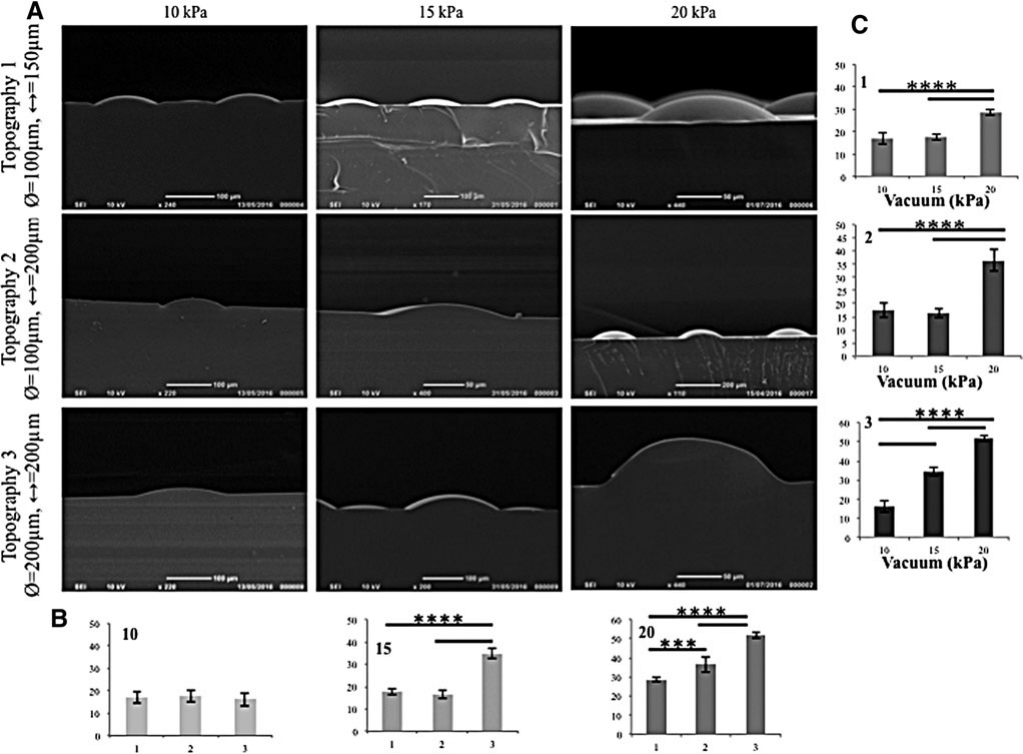
Vacuum-induced indentations. **(A)** SEM of PDMS stamp showing PLGA deformation by vacuum pressure. **(B, C)** ImageJ was used to quantitate deformation as a function of topography (1–3) (B) and vacuum pressure (10, 15, or 20 kPa) **(C)**. *y*-Axes in **(B, C)** are depth of deformation (μm). *n* = 3 images per sample; *n* = 3 samples for each condition. ****p ≤ 0.0001 for all comparisons indicated, except ***p < 0.001. PDMS, polydimethylsiloxane.

The PLGA membrane and template were assembled in a rig made from transparent acrylic (Fig. 3A–D and Supplementary Movie S1). Then a vacuum was applied through two tubes coming from both sides of the rig and connecting to a vacuum pump (Fig. 3E). The pressure from the vacuum drew the membrane through the template, creating invaginations varying in depth and spacing according to topography and applied vacuum pressure (Fig. 4).

To quantify the degree of membrane deformation achieved, PDMS was poured on top of the deformed membrane and then left to cure, as described previously,^18^ creating an imprinted stamp of the deformed membrane. The PDMS stamps were imaged using SEM (Fig. 4A), and the images were analyzed using ImageJ. Vacuum pressures of 10, 15, and 20 kPa were used to deform the membrane. Quantitative analysis confirmed that an increase in vacuum pressure correlated with an increased depth of membrane topographies. Under 15 and 20 kPa (Fig. 4B, C) vacuum pressures, there was a statistically significant difference in the deformation of topography 3 compared to topographies 1 and 2. Regardless of topography there was a statistical difference between the deformation due to 20 kPa and the other two vacuum pressures (Fig. 4B, C). For subsequent experiments, a vacuum pressure of 20 kPa was selected because it gave the highest degree of deformation.

### Optimizing keratinocyte adhesion to the PLGA membrane

**FIG. 5.**
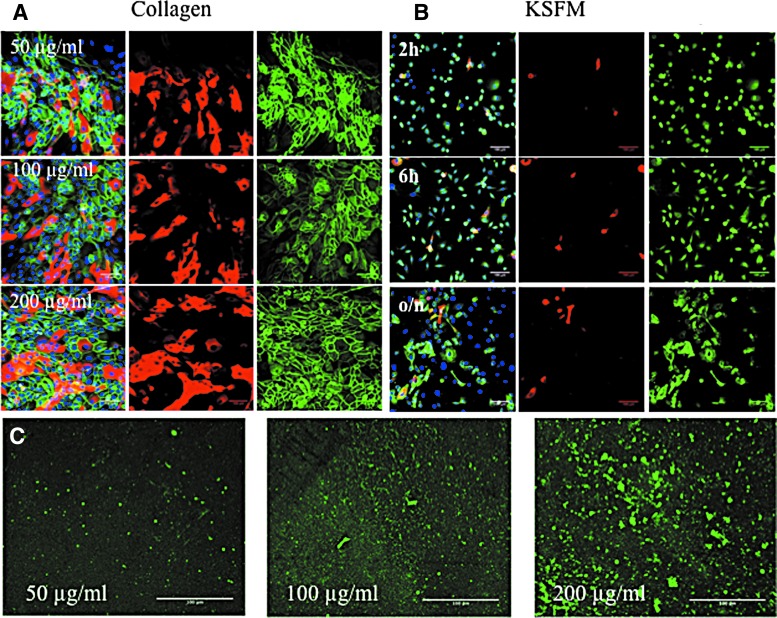
Cell adhesion on flat collagen-coated PLGA. **(A)** Effect of different collagen coating concentrations. PLGA was coated with 50, 100, or 200 μg/mL collagen type I, and keratinocytes were seeded at a density of 75,000 cells/cm^2^ for 48 h in FAD medium. **(B)** Cell attachment for different times. Keratinocytes were seeded at a density of 75,000 cells/cm^2^ in KSFM for 2 h, 6 h, or overnight (o/n). **(A, B)** Cells were fixed and labelled with antibodies to involucrin (*red*), β1 integrin (*green*), and DAPI as a nuclear counterstain (*blue*). *Left hand panels* show merged images of the fields on the *right*. **(C)** PLGA membranes were coated with the collagen concentrations shown and imaged by confocal microscopy. FAD, F12, adenine, and DMEM; KSFM, keratinocyte serum free medium. Scale bars are 100 microns.

To determine the optimal collagen coating concentration for the PLGA membrane, keratinocytes were seeded at a density of 75,000 cells/cm^2^ on concentrations of 50, 100, or 200 μg/mL bovine type I collagen and cultured in complete FAD medium for 48 h. The cultures were then fixed and stained with antibodies to β1 integrin and involucrin with DAPI as a nuclear counterstain. As shown in Figure 5A, the cells formed stratified sheets of basal (β1 integrin-positive) and suprabasal, differentiating (involucrin-positive) cells on each collagen coating. Therefore, a concentration of 50 μg/mL was selected for subsequent experiments because it was sufficient to support keratinocyte attachment.

We also evaluated the optimal plating time to allow stratified sheet formation before applying the vacuum (Fig. 5B). Regardless of whether cells were seeded in complete KSFM (Fig. 5B) or complete FAD (data not shown), the cells that adhered up to 6 h were primarily β1 integrin-positive involucrin-negative cells, consistent with our previous studies.^18^ However, by 24 h involucrin-positive cells were present, indicating that some cells had initiated terminal differentiation. For subsequent experiments, cells were seeded overnight in KSFM and then transferred to complete FAD medium at the time when the vacuum was applied. Figure 5C shows the deposition of collagen onto the PLGA membranes, as evaluated by immunofluorescence labelling.

### Stem cell patterning on dynamic topographies

**FIG. 6.**
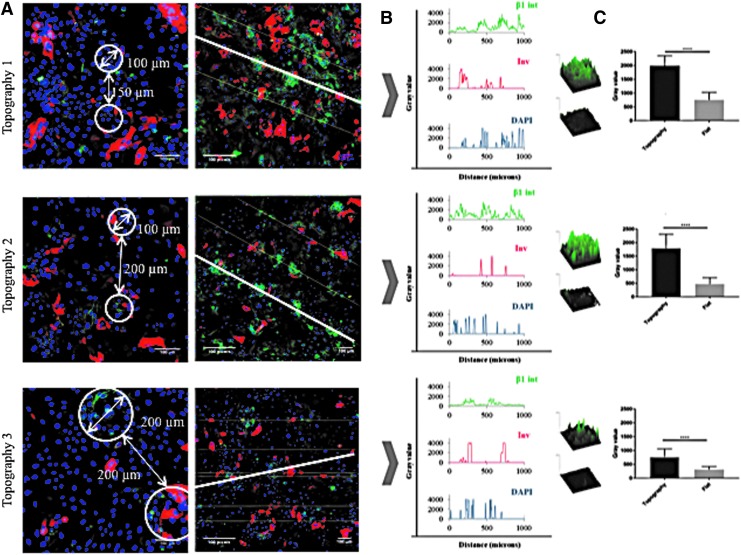
Effect of topographies on involucrin and β1 integrin expression after culture under vacuum for 48 h. Cells were fixed and labelled with antibodies to involucrin (*red*), β1 integrin (*green*), and DAPI as a nuclear counterstain (*blue*). **(A)** Representative high magnification images showing spacing of holes (*circled*) (*left hand column*) and lower magnification views (*right hand column*) showing the *lines* through the center of adjacent holes that were used to measure corresponding pixel intensities in **(B)**. **(C)** Images show representative examples of β1 pixel intensity per indented versus flat area. Histograms show pixel intensity per 120 pixels total for topographies 1 (*top*) and 2 (*middle*) (equivalent to 100 × 100 μm area) and 240 pixels total for topography 3 (*bottom*) (equivalent to 200 × 200 μm area). *n* = 3 flat and 3 indented regions per membrane and 3 membranes. ****p ≤ 0.0001. Scale bars are 100 microns.

After 48 h of applying a constant 20 kPa vacuum pressure, PLGA membranes were recovered from the rig and the attached cells were fixed, immunostained, and examined by confocal microscopy. Although the pressure had been removed, the position of the indentations, corresponding to the holes in the template, was readily observed (Fig. 6A). By drawing a line through the center of each hole (Fig. 6A), we could quantitate integrin and involucrin expression relative to the topographies on the basis of pixel intensity (*y*-axis) per unit length (*x*-axis) (Fig. 6B). On all three topographies, β1 integrin bright cells clustered in the holes, but in contrast patterning of involucrin-positive cells was not observed. Topography 3 differed from topographies 1 and 2 in that there was a ring of integrin-bright cells at the periphery of each hole, rather than a uniform distribution of integrin-bright cells across the entire hole diameter (Fig. 6A, B). There was no clustering of β1 integrin bright or involucrin-positive cells on the flat membrane controls. To compare the total fluorescence signal in individual indented versus flat regions of the same topographical feature, pixel intensity per unit area was measured (Fig. 6C). We quantitated three indented and three flat regions per well in a total of three wells per topography. On all three topographies the fluorescence intensity in the indentations was significantly higher than in the flat regions (Fig. 6C).

### Role of intercellular adhesion in stem cell patterning

**FIG. 7.**
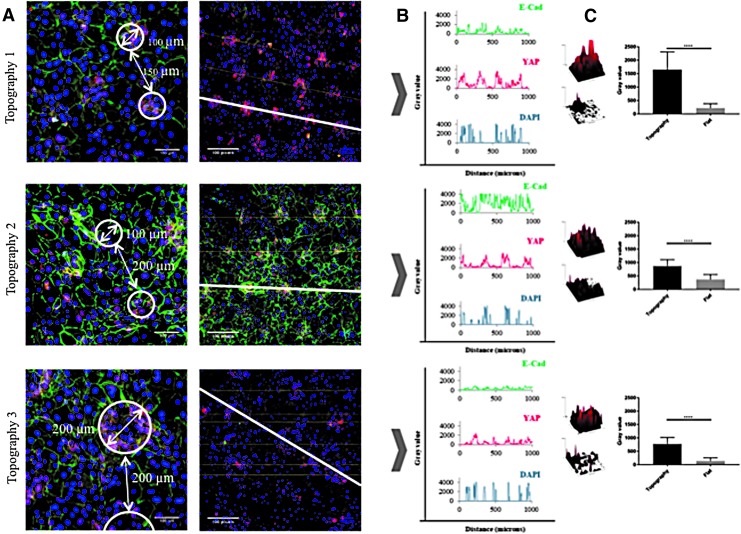
Effect of topographies on YAP and E-cadherin expression after culture under vacuum for 48 h. Cells were fixed and labelled with antibodies to YAP (*red*), E-cadherin (*green*), and DAPI as a nuclear counterstain (*blue*). **(A)** Representative high magnification images showing spacing of holes (*circled*) (*left hand column*) and lower magnification views (*right hand column*) showing the *lines* through the center of adjacent holes that were used to measure corresponding pixel intensities in **(B)**. **(C)** Images show representative examples of YAP pixel intensity per indented versus flat area. Histograms showing pixel intensity per 120 pixels total for topographies 1 (*top*) and 2 (*middle*) (equivalent to 100 × 100 μm area) and 240 pixels total for topography 3 (*bottom*) (equivalent to 200 × 200 μm area). *n* = 3 flat and 3 indented regions per membrane and 3 membranes. ****p ≤ 0.0001. Scale bars are 100 microns.

We have previously observed that the YAP/TAZ pathway is activated in stem cell clusters on undulating PDMS topographies, as evidenced by nuclear accumulation of YAP.^23^ On the PDMS substrates, YAP activation is dependent on intercellular adhesion and can be prevented by pharmacological inhibition of Rho kinase. This prompted us to examine the localization of E-cadherin and YAP in cells on the dynamic substrates (Fig. 7).

**FIG. 8.**
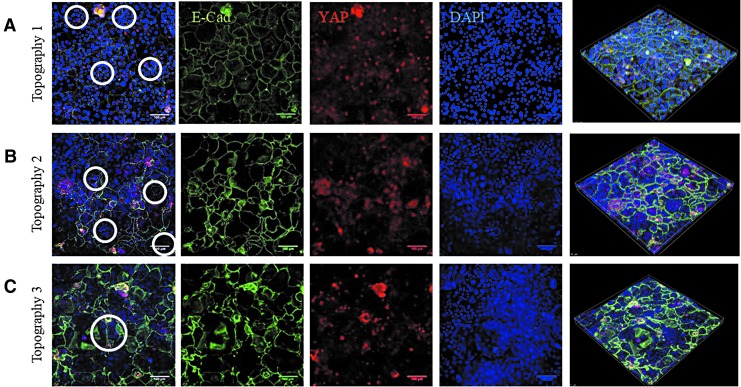
Effect of Rho kinase inhibition on YAP and E-Cadherin expression. Representative images of topographies 1 **(A)**, 2 **(B)**, and 3 **(C)** after culturing keratinocytes for 48 h under vacuum pressure in FAD medium containing ROCK inhibitor. Cells were fixed and labelled with antibodies to YAP (*red*), E-cadherin (*green*), and DAPI as a nuclear counterstain (*blue*). Individual confocal images and Z-stacks (*right hand side*) are shown. ROCK, Rho kinase. Scale bars are 100 microns.

Immunostaining showed that in the indentations of all three topographies, YAP localized to the cell nucleus, whereas elsewhere most cells had cytoplasmic YAP (Fig. 7A). As in the case of β1 integrin-bright cells (Fig. 6), the cells with nuclear YAP on Topography 3 formed a ring at the periphery of each feature (Fig. 7A). E-Cadherin immunostaining was clustered in the invaginations on the topographies (Fig. 7A, B). On all three topographies the fluorescence intensity of YAP in the indentations was significantly higher than in the flat regions (Fig. 6C). Treatment with the Rho kinase inhibitor Y-27632 (Enzo ALX-270-333; 10 μM) at the start of application of vacuum pressure prevented the patterning of E-cadherin and nuclear YAP on all three topographies (Fig. 8).

## Discussion

In this study we describe the creation of a rig in which dynamic undulations of the epidermal–dermal junction can be created as a platform to study changes in the skin with age and disease. It was first reported over 20 years ago that stem cells in adult human epidermis are patterned with respect to the undulations of the epidermal–dermal junction.^24^ However, until recently the underlying mechanisms were unclear. The clustering of integrin-bright keratinocytes on the tips of PDMS substrates that mimic the topography of healthy young skin (feature diameter of 150 μm and center-to-center distance of 100 μm)^18^ indicated that clustering is not dependent on the presence of other cell types, such as fibroblasts or vascular endothelial cells. This is also consistent with the finding that keratinocytes can spontaneously organize into clusters when cultured on a flat substrate.^24^ We have also shown that clustering is dependent on intercellular adhesion and can be disrupted by inhibiting Rho kinase signaling or the nonmuscle myosin II inhibitor Blebbistatin.^23^

Our observations with the dynamic rig are largely consistent with the earlier studies on static PDMS substrates,^18^ specifically that clustering can be achieved based on topography and that it is dependent on Rho kinase activity. However, it also provides new insights into the process. First, we show that topographical features can impose patterning on a flat sheet of cells and that the reorganization occurs within 48 h. Second, although *in vivo* lineage tracing in mouse skin has established that differentiating cells tend to be the progeny of basal layer cells that lie directly beneath them,^25^ we found that stem cell clustering can be induced independent of the location of differentiating involucrin-positive cells. This is consistent with the finding that differentiating cells can move relative to underlying basal cells, for example during wound healing.^26–28^

One surprising finding was that integrin-bright clusters formed in the indentations, rather than the tips, of the features of dynamic substrates. This is the opposite orientation to that found on static topographies.^18^ However, it is in agreement with the observation that in some body sites stem cells are located in the rete ridges.^24,29^ While further work is required to uncover the underlying mechanisms, one interpretation of our findings is that it is the undulations rather than their direction that is important in determining stem cell patterning. Forces exerted through intercellular adhesion may differ according to the slope of the undulations. A further possibility is that patterning of stem cells depends on whether they are seeded directly onto an undulating surface^18^ or whether undulations are imposed on a flat cell sheet. This is an interesting possibility in situations in which epidermal–dermal topology changes over time, for example, in the development of psoriatic lesions.^6^

We observed that on Topography 3, which has the largest diameter holes, the integrin bright cells with nuclear YAP formed a rig at the edge of the holes rather than being uniformly distributed. This suggests that local forces at the edge of the features are most important and correlate with the organization of intercellular adhesions.^30–32^ Crowding in the epidermal basal layer is known to affect cell shape and play a role in triggering exit into the suprabasal layer through a decrease in cortical tension and increased cell–cell adhesion.^30^ We envision that future modifications to the rig to allow live imaging may reveal whether or not the cells in the center of Topography 3 are more likely to differentiate than cells are the periphery.^23^

In conclusion, we have designed, developed, and optimized a novel device that provides a better understanding of how stem cell behavior is influenced by the topography of the epidermal–dermal junction. The strength of the design is that it imposes dynamic changes in topography. The specific regions where stem cells are located can be controlled in a dynamic model that allows us to simultaneously mimic different topographical features by applying vacuum pressure through a rig.

## Supplementary Material

Supplemental data

## Data Availability

The raw/processed data required to reproduce these findings cannot be shared at this time due to technical or time limitations.
